# Brain Architecture of Punishment Learning

**DOI:** 10.1523/JNEUROSCI.0925-26.2026

**Published:** 2026-08-10

**Authors:** Alexandra V. Gregory, James Diefenbach, Eun A. Choi, Jason Soo, Jessica Chen, A. Simon Killcross, Philip Jean-Richard-dit-Bressel, Gavan P. McNally

**Affiliations:** School of Psychology, UNSW, Sydney, New South Wales 2052, Australia

**Keywords:** amygdala, chemogenetics, Fos, learning, network, punishment

## Abstract

Learning from punishment allows animals to suppress actions that produce adverse consequences while maintaining other rewarded behaviors. However, the brain mechanisms of this learning are poorly understood. Here, we combined instrumental behavioral analysis, whole-brain Fos mapping, spatial transcriptomics, computational network analysis, and chemogenetic inhibition in male and female mice. A within-subject yoking procedure showed that suppression depended on the instrumental response–punisher contingency rather than matched shock exposure or embedded pavlovian stimulus–shock relations. Whole-brain Fos network analysis showed marked reorganization of brain-wide Fos correlation structure after punishment learning. Punishment preserved modular, small-world organization characteristic of brain networks while reallocating regional community membership and increasing the centrality of the basolateral amygdala (BLA), zona incerta (ZI), and midbrain tegmentum. Spatial transcriptomics within these regions identified punishment-associated transcriptional programs in BLA glutamatergic neurons, ZI GABAergic neurons, and multiple ventral midbrain GABAergic and dopaminergic populations. In silico deletion predicted that the BLA, ZI, and rostral linear nucleus jointly support punishment learning. Consistent with this, multisite chemogenetic inhibition of these regions impaired punishment learning. Single-region inhibition revealed dissociable contributions of BLA and ZI to within-session and between-session retention of punishment learning. Together, these findings show that punishment learning is supported by a brain network that enables animals to selectively suppress actions that produce adverse consequences.

## Significance Statement

Punishment learning is essential for adaptive behavior because it allows animals to stop actions that produce harm while maintaining other rewarded actions. We show that this form of learning is not explained by shock exposure, pavlovian fear, or activation of a single brain region. Instead, punishment learning reorganizes brain-wide activity networks, recruits spatially structured transcriptional programs in specific neuronal populations, and depends on the function of the basolateral amygdala, zona incerta, and rostral linear nucleus of the raphe. These findings provide a multiscale account of how the brain learns from adverse consequences.

## Introduction

Learning to modify behavior in response to adverse consequences is essential for adaptive decision-making. When an action produces harm, animals must use this information to suppress that action while maintaining other behaviors that earn reward (Jean-Richard-Dit-Bressel et al., 2018). This capacity is central to survival, and disruptions in this capacity have been linked to many forms of maladaptive behavior (McNally et al., 2023). Much of what is known about aversive control comes from pavlovian fear paradigms, in which external cues elicit defensive responses (McNally et al., 2011; Herry and Johansen, 2014). These paradigms have been highly influential, but they do not explain how animals learn that their own actions cause aversive outcomes or how this learning is used to regulate action selection.

Instrumental punishment learning poses a distinct problem. It requires the animal to detect a response–punisher contingency, integrate that adverse consequence with ongoing reward, and selectively suppress the punished action without producing general behavioral inhibition (Bolles et al., 1975, 1980). Reinforcement-learning models often describe punishment as the negative counterpart of reward, in which aversive outcomes reduce action value and thereby weaken responding (Sutton and Barto, 2018). However, punishment is not simply the opposite of reward (Mackintosh, 1983) and does not simply weaken responding (Marchant et al., 2013; Bouton and Broomer, 2023). Punishment is likely to require coordinated changes across systems that encode aversive value, represent actions, integrate sensory and motivational information, and implement behavioral control.

The amygdala is an important candidate for this process. Humans (Bechara et al., 2003) or rodents (Killcross et al., 1997) with amygdala damage are impaired in punishment learning. Rodent lesion (Killcross et al., 1997), pharmacological (Jean-Richard-Dit-Bressel and McNally, 2015), chemogenetic (Piantadosi et al., 2026), and recording (Jean-Richard-dit-Bressel et al., 2022; Piantadosi et al., 2026) studies assign this role to the amygdala's basolateral nucleus. However, selectively suppressing a punished action requires animals to integrate punishment with ongoing reward, regulate action selection, and maintain alternative actions. This suggests that the basolateral amygdala (BLA) may operate as one component of a broader punishment learning network. The identity of this network, the molecularly defined cell populations recruited within its key nodes, and the causal contribution of those nodes to punishment learning remain unknown.

Here we combined instrumental behavioral analysis, whole-brain Fos mapping, graph-theoretic network analysis, spatial transcriptomics, in silico network perturbation, and chemogenetic inhibition to define the neural architecture of instrumental punishment learning. First, we used a within-subject yoking procedure to distinguish instrumental response–punisher learning from embedded pavlovian stimulus–shock relations. We then mapped brain-wide Fos expression after punishment learning and asked whether punishment altered regional activity or the coordinated organization of activity across the brain. Network analysis identified a punishment network centered on hubs in the BLA, zona incerta (ZI), and midbrain tegmentum. Spatial transcriptomics within these hubs identified punishment-associated transcriptional programs in molecularly defined neuronal populations. Finally, in silico deletion and chemogenetic inhibition tested whether these hubs were necessary for punishment learning. Together, these experiments show that instrumental punishment learning is supported by a distributed network that enables animals to selectively suppress actions that produce adverse consequences.

## Materials and Methods

### Subjects

Male and female C57BL/6J mice (Australian Resources Centre; 8–10 weeks old) were housed in ventilation racks on corn cob bedding in a climate-controlled colony room under a 12:12 h light/dark cycle. Although both sexes were used, these experiments were neither designed nor powered to assess sex differences. Mice had *ad libitum* access to food and water until 2 d prior to commencement of behavioral training. For the remainder of the experiment, they received 1–2 g of food and *ad libitum* access to water each day. Experiments were approved by the UNSW Animal Care and Ethics Committee and performed in accordance with the Animal Research Act 1985 (NSW), under both ARRIVE guidelines and the National Health and Medical Research Council Code for the Care and Use of Animals for Scientific Purposes in Australia (2013).

### Behavioral apparatus

All behavioral training and testing occurred in ENV-307A Standard Modular Chambers for Mouse (18 × 15 × 15 cm; Med Associates). Each chamber was fitted with two retractable stainless-steel levers located on either side of the magazine for delivery of 20 mg grain pellets (BioServ, Dustless Precision Pellets). The chambers had a stainless-steel grid floor through which the punisher was delivered. Each chamber was located inside a light- and sound-attenuating compartment. Any contact that was able to depress the lever was recorded as a lever press.

### Surgeries and viral injections

Mice underwent stereotaxic surgery for intracranial delivery of adeno-associated viral vectors. Mice were anaesthetized using 5% isoflurane in oxygen-enriched air and then fixed into a stereotax (Model 1900, David Kopf Instruments). Mice were maintained on 2.5% isoflurane during surgery. A 0.1 ml Marcaine (0.5%) was injected subcutaneously at the incision site. Ophthalmic gel (Viscotears, Alcon) was applied to avoid eye-drying.

AAV5-hSyn-hM4D(Gi)-mCherry and AAV5-hSyn-mCherry were used. pAAV-hSyn-hM4D(Gi)-mCherry was a gift from Bryan Roth (Addgene viral prep #50475-AAV5; http://*n*2t.net/addgene:50475; RRID:Addgene_50475). pAAV-hSyn-mCherry was a gift from Karl Deisseroth (Addgene viral prep #114472-AAV5; http://n2t.net/addgene:114472; RRID:Addgene_114472). For triple silencing, 0.2 µl was microinjected into BLA (coordinates in millimeter relative to the bregma; −2.05 anteroposterior; ±3.30 mediolateral, and –5.0 dorsoventral), 0.1 µl into rostral ZI (rostral 1.0 anteroposterior, ±0.75 mediolateral, −4.60 dorsoventral), and 0.15 µl into caudal ZI (−2.3 anteroposterior, ±1.4 mediolateral, and –4.50 dorsoventral). A 0.15 µl was injected into the rostral linear nucleus of the raphe (RLi; coordinates relative to the lambda; ±0.0 mediolateral, −0.65 anteroposterior, and –4.05 dorsoventral). For single-region silencing, the same coordinates and volumes were used. All infusions were made via 32 gauge conical-tipped microinfusion syringe (SGE Analytical Science) connected to a UMP3 with SYS4 microcontroller microinjection system (World Precision Instruments) over a 5 min period. The needle remained in place for 5 min. Mice received subcutaneous injections of antibiotic (Duplocillin, 0.15 ml/kg of body weight, s.c.) and 5 mg/kg carprofen (Rimadyl, Zoetis) immediately postsurgery. Mice had at least 7 d recovery from surgery.

### Behavioral procedures

#### Experiment 1: within-subject yoking

Mice first received two sessions of fixed-ratio (FR) 2 lever-press training. Both levers were extended, and every second press on either lever was reinforced with delivery of a pellet to an external magazine. Each lever retracted after being pressed 25 times. The session ended after 50 min or when both levers had retracted. Mice then received daily 40 min lever-press training sessions over the next 8 d. Left and right levers were alternately presented individually for 5 min at a time. There were four trials per lever across the 40 min session. Lever pressing was reinforced with pellet delivery on a variable-interval 30 s (VI30) schedule. The first lever to be extended (left or right) was fully randomized. Lever-press training was conducted until a threshold of at least 12 LP/min per lever and equivalent pressing on each lever was reached.

Mice next received a 40 min session of yoked training. Left and right levers were alternately presented for 5 min. There were four trials per lever across 40 min. The first lever to be extended (left or right) was fully randomized. The first lever presented, designated “instrumental,” was rewarded on a VI30 schedule and punished (0.5 s, 0.3 mA) on an FR8 schedule. After 5 min, the instrumental lever was retracted, and the second lever, designated “pavlovian,” was inserted. This lever was also rewarded on a VI30 schedule and 0.5 s; 0.3 mA footshocks were delivered in a response noncontingent manner. Each mouse received the same number and timing of footshocks as it had received during its immediately preceding instrumental trial.

#### Experiment 2: whole-brain Fos

After lever-press training (see above), mice received a single session of training. For group Control (*n* = 8), this 40 min session was identical VI lever-press training. For group Punishment (*n* = 8), this session was identical to the VI30 lever-press training sessions, except responses on one of the levers (designated punished lever) delivered a 0.5 s shock on a FR8 schedule independent of pellet outcome. The intensity of the punisher was 0.3 mA. The punished lever (left or right) was counterbalanced across animals. The other lever (designated unpunished lever) was rewarded by pellets but not punished by shocks. Which lever was presented first was counterbalanced across animals.

After a single session of punishment (0.3 mA, 0.5 s footshock), mice were killed 90 min via an intraperitoneal injection of 0.1 ml sodium pentobarbitone (100 mg/kg). Mice were then transcardially perfused with 10–20 ml ice-cold saline (prewash), followed by 20 ml 4% paraformaldehyde (PFA) in phosphate-buffered saline (PBS). Brains were extracted and postfixed in 4% PFA at 4°C for 24 h, rinsed twice in PBS, and stored long-term in PBS containing 0.02% sodium azide at 4°C until further processing.

PFA-fixed samples were preserved using SHIELD reagents (LifeCanvas Technologies) using the manufacturer's instructions. Samples were delipidated using LifeCanvas Technologies Clear + delipidation reagents. Following delipidation, samples were labeled using eFLASH (Yun et al., 2019) technology which integrates stochastic electrotransport (Kim et al., 2015) and SWITCH (Murray et al., 2015) using a SmartBatch+ (or SmartLabel) device (LifeCanvas Technologies). After immunolabeling (primary, rabbit anti-C-Fos antibody, 3.5 µg, sample; secondary, donkey anti-rabbit Setau-647, 5.25 µg per sample), samples were incubated in 50% EasyIndex (RI = 1.52, LifeCanvas Technologies) overnight at 37°C followed by 1 d incubation in 100% EasyIndex for refractive index matching. After index matching, the samples were imaged using a SmartSPIM axially swept light-sheet microscope using a 3.6× objective (0.2 NA; LifeCanvas Technologies).

Samples were registered to the Allen Brain Atlas (Allen Institute, https://portal.brain-map.org/) using an automated process (alignment performed by LifeCanvas Technologies). A propidium iodide channel for each brain was registered to an average atlas (generated by LCT using previously registered samples). Registration was performed using successive rigid, affine, and b-spline warping algorithms (SimpleElastix, https://simpleelastix.github.io/).

Automated cell detection was performed by LifeCanvas Technologies using a custom convolutional neural network created with the Tensorflow python package (Google). The cell detection was performed by two networks in sequence. First, a fully convolutional detection network (https://arxiv.org/abs/1605.06211v1) based on a U-Net architecture (https://arxiv.org/abs/1505.04597v1) was used to find possible positive locations. Second, a convolutional network using a ResNet architecture (https://arxiv.org/abs/1512.03385v1) was used to classify each location as positive or negative. Using the previously calculated Atlas Registration, each cell location was projected onto the Allen Brain Atlas in order to count the number of cells for each atlas-defined region.

#### Experiment 3: spatial transcriptomics

After lever-press training (see above), mice received a single session of training. For group Control (*n* = 4), this 40 min session was identical VI lever-press training. For group Punishment (*n* = 4), this session was identical to the VI30 lever-press training sessions, except responses on one of the levers (designated punished lever) delivered a 0.5 s shock on a FR8 schedule independent of pellet outcome. The intensity of the punisher was 0.3 mA. The punished lever (left or right) was counterbalanced across animals. The other lever (designated unpunished lever) was rewarded by pellets but not punished by shocks. Which lever was presented first was counterbalanced across animals.

Immediately afterward, mice were anaesthetized with isoflurane and then killed. Heads were submerged in ice-cold water, and brains were rapidly extracted. The extracted tissue was immediately flash-frozen in liquid nitrogen and stored at –80°C until further processing. The 14 μm coronal sections were prepared using a cryostat and mounted onto a MERSCOPE slide (lot numbers 24060315, 24081215) and handled according to manufacturer guidelines (MERSCOPE User Guide—Fresh and Fixed Frozen Tissue Sample Preparation—91600002 Rev B). Each slide contained three sections from a punished animal and three sections at the same coronal level from a control animal. Sections were chosen to span the anterior–posterior boundary of the target regions. Slides remained at −20°C for at least 5 min prior to 15 min incubation in fixation buffer at room temperature for 15 min. Slides were then washed three times with 5 ml 1× PBS (5 min each) before application of 5 ml 70% ethanol and overnight storage at 4°C to permeabilize the tissue. Slides were stored at 4°C prior to hybridization.

MERFISH using the MERSCOPE Gene Panel type, PanNeuro 500 gene mouse (catalog #20300123), was performed according to manufacturer instructions; autofluorescence quenching was not used. Postsectioning the sections were kept at −20°C for 20 min followed by 15 min incubation in 4% PFA, washed three times for 5 min with RNAse-free PBS, and were kept in 70% ethanol to permeabilize the tissue. Following 5 d at 4°C in 70% v/v ethanol, tissues were washed in sample prep wash buffer (Vizgen) and incubated in formamide buffer (Vizgen) at 37°C for 30 min. After 50 μl of the encoding probe mixture was applied directly to the tissue, the tissue was covered with parafilm and incubated for 48 h at 37°C in a humidified chamber. Following hybridization, samples were incubated in formamide buffer at 47°C for 30 min, washed with sample prep wash buffer, and incubated for 1 min in gel embedding solution [5 ml gel embedding premix (Vizgen), 25 µl 10% APS, 2.5 µl TEMED]. The tissue was embedded in a thin layer of polyacrylamide gel using inverted embedding technique. Embedded tissues were then cleared in 5 ml of clearing solution [5 ml clearing premix (Vizgen), 50 µl Proteinase K (NEB)] for up to 24 h at 37°C, until the tissue was optically transparent. Cleared samples were washed in sample prep wash buffer, stained with DAPI and PolyT (Vizgen) for 15 min at RT, and incubated in formamide for 10 min prior to imaging. Imaging followed the Merscope Instrument Preparation guide (number 91500001) with 10 mm section thickness selection and MERSCOPE 500gene imaging kit (10400006). For each slide, a 1 cm^2^ ROI was manually drawn based on DAPI and PolyT stains (10× magnification). Cell and transcript segmentation was completed via Vizgen Post-processing tool (VPT) using DAPI and PolyT–Cellpose model with Gaussian filter set to 30 and cell size 30. One midbrain slide was damaged during reactions, so *n* = 3 mice per group were used for midbrain analyses.

#### Experiments 4 and 5: chemogenetic inhibition

Mice had received stereotaxic infusion of AAVs encoding hM4Di or mCherry control (see above). Mice received lever-press training as described above. They received a dummy intraperitoneal injection on the final day of VI lever-press training to acclimatize them to injections. Next, mice received punishment training. Punishment was identical to the VI30 lever-press training sessions, except responses on one of the levers (designated punished lever) delivered a 0.5 s shock on a FR8 schedule independent of pellet outcome. The intensity of the punisher was 0.3 mA. The punished lever (left or right) was counterbalanced across animals. The other lever (designated unpunished lever) was rewarded by pellets but not punished by shocks. Which lever was presented first was counterbalanced across animals. Thirty minutes prior to the punishment session, all mice [hM4D(Gi) and mCherry virus] received an intraperitoneal injection of CNO (3 mg/kg, 0.15 ml, 0.5% v/v DMSO). Forty-eight hours later, they received a test of retention of punishment learning, identical to the VI30 training sessions. The first 5 min block of each lever presentation was used as test data.

Next, they received 2–3 d of VI30 lever-press training to equate responding on the levers. Then, all mice received a 0.15 ml intraperitoneal vehicle injection 30 min prior to another punishment session which was otherwise identical to the first (i.e., test order CNO vs vehicle was not counterbalanced). Vehicle injections consisted of 0.5% v/v DMSO. Finally, mice received a second test session 48 h later, identical to the first test session.

### Histology and immunohistochemistry

At the end of the experiment, mice were deeply anaesthetized via intraperitoneal sodium pentobarbital (100 mg/kg) and perfused with 0.9% saline solution containing 1% sodium nitrite and heparin (5,000 IU/ml), followed by phosphate buffer solution (0.1 M) with 4% PFA. Brains were extracted and stored in 4% PFA solution for 24 h before being placed in 20% sucrose in PBS until slicing. Brains were sliced coronally (40 µm) into five sequential series. Slices were stored in 0.1% sodium azide until they were mounted on 4% gel glass microscope slides, coverslipped and imaged. To verify AAV injection sites, natural fluorescence was imaged on a Zen Axioscan Slide Scanner at 10× magnification. Two channels were used: AF 594 to visual virus expression and AF488 to visual anatomical landmarks.

### Experimental design and statistical analysis

#### Behavior

The primary behavioral variable of interest was preference (suppression) ratio calculated as *a*/(*a* + *b*) where *a* is the number of punished lever presses and *b* is the number of unpunished lever presses. Values range from 0 to 1, where 0.5 indicates no lever preference and values <0.5 indicate lower preference for punished lever relative to unpunished lever. Each session was divided into four 10 min blocks, where mice experience 5 min with each lever individually. Suppression ratio for each block was calculated from the lever presses in this 10 min window, responding on the punished lever in 5 min, divided by the total number of lever presses in the entire 10 min window. These data were analyzed via ANOVA, and the Type I error rate (*α*) for all analyses was controlled at 0.05. These analyses were conducted using GraphPad Prism version 11.0.0 and the Psy statistical package.

#### Whole-brain Fos

Analyses were conducted in Python 3.10 using NetworkX (v2.8) and associated libraries (NumPy, Pandas, Matplotlib/Seaborn, python-louvain). First, regional group differences were tested independently across 206 brain regions. Multiple-comparison correction was performed using the Benjamini–Hochberg false discovery rate procedure across the full set of regional tests. Corrected effects were considered significant at FDR-adjusted *p* < 0.05. Uncorrected *p* values are provided for transparency but were not used to define corrected statistical significance.

Next, for each group, Pearson’s correlation matrices of total cell densities (Fos+ cells/mm^3^) across regions were computed and unweighted functional networks constructed by thresholding absolute correlations at *r* = 0.80 (diagonals set to zero) and binarizing edges.

Communities were identified with the Louvain algorithm (resolution *γ* = 1.0). To obtain robust partitions, we performed 200 independent runs with distinct random seeds (0–199) and formed a coassignment matrix representing the probability that two nodes clustered together across runs. Edges with weight ≥*τ* = 0.60 were retained, and Louvain was rerun once on this thresholded graph to define consensus communities. Node stability was quantified as the proportion of runs in which each node's label matched its consensus assignment. Partition similarity between groups was quantified using the Adjusted Rand Index and Normalized Mutual Information and a Jaccard similarity matrix was used to visualize cross-group module correspondence.

Node-level centrality included degree, betweenness, and closeness (Bullmore and Sporns, 2009; Basset and Sporns, 2017; Faskowitz et al., 2022). To compare regional importance across groups, we *Z*-scored degree centrality values within each network and then computed the difference in *Z*-scores between Punishment and Control groups. This identified regions whose relative centrality increased or decreased under punishment. Network-level metrics included degree distributions, degree assortativity, clustering, characteristic path length, global efficiency, and diameter. Small worldness (*σ*) was computed as *σ* = (C/C_rand)/(L/L_rand), using 200 degree-preserving rewired surrogates (seed = 42). Characteristic path length was evaluated on the largest connected component (LCC).

Robustness was assessed by targeted deletion: nodes were ranked by betweenness centrality and removed sequentially while tracking the LCC fraction to yield percolation curves. To test the contribution of three hubs that increased in centrality under punishment (ZI; RLi; posterior BLA), we recalculated topology and global communicability after removing each hub individually and all three together. Communicability was defined as the sum of all pairwise values from the matrix exponential of the absolute adjacency (off-diagonal). Specificity was assessed by generating a null distribution of 5,000 random triple deletions (punished network), from which a permutation *p* value was computed.

#### Spatial transcriptomics

The MERFISH expression matrix was imported to Vizgen Merscope Visualizer and selected regions exported for further data analysis. First, cell-type annotation was performed using Allen Institute's MapMyCells tool. For each sample, the AnnData file from Vizgen viewer was uploaded to the MapMyCells web interface, to return a table containing subclass_name, subclass_bootstrapping_probability, supertype_name, and supertype_bootstrapping probability. These annotations were imported into the MERSCOPE Visualizer to guide manual delineation of the amygdala and subthalamic–hypothalamic zone. Polygon coordinates and corresponding AnnData (.hdf5) files were exported, and all cells within the selected regions were extracted using custom Python scripts. The VTA region was automatically selected using a custom script that created a convex hull containing 97% of all cells (chosen by Mahalanobis distance) with “VTA” in the subclass name. Similarly, the “RL” cells were selected by a similar method. These regions were manually verified.

Cells with total gene-expression counts below the 5th percentile or above the 98th percentile for each slide were excluded. Expression values were normalized to a total count of 5,000 per cell, a threshold chosen to exceed the median total counts for the BLA while minimizing information loss. A UMAP plot of cells showed no visual clustering by slide, and thus batch correction was not performed (Fig. S1). The genes with the 100 lowest variances were calculated for each region and anterior–posterior combination and removed to prevent noise from very low variance genes affecting the results. The data were then passed into the consensus non-negative matrix factorization (cNMF) package where gene expression was variance scaled so that genes on different expression scales would have equal weighting in the program calculation.

cNMF (Kotliar et al., 2019) was used to identify transcriptional programs. For each brain region and condition (control or punished), a principal component analysis scree plot showing log10 variance explained show an elbow ∼10–19 programs for all regions. Accordingly, cNMF models were fit using 10–20 programs. Plots of reconstruction error and stability, together with principal component analysis scree plots showing log₁₀ variance explained, were used to select an optimal number of programs (n_programs) that maximized stability while minimizing error. The number of programs was kept constant between conditions when possible, except when this would cause a relatively unstable solution for a condition, in which case the number was allowed to differ by a maximum of 2 (although in practice control and punished never differed by more than one). When ambiguous, a slightly higher n_programs was chosen to ensure adequate resolution of transcriptional diversity.

For each region–condition combination, cNMF output included the top-weighted genes and cell-level program usages. Jaccard similarity matrices were computed to identify candidate punishment-specific programs. To assess robustness, leave-one-out resampling was performed by sequentially excluding each sample and recomputing programs for both conditions, yielding *n* program solutions per condition. Candidate punishment-specific programs were retained only if (1) no control leave-one-out run recapitulated them (Jaccard Index ≥0.5) and (2) all punished leave-one-out runs did (Jaccard Index <0.5 indicating a failure to reproduce). For the remaining punishment-specific programs, the dominant MapMyCells subclasses (those with usages in the top 30 of all subclasses) contributing to each program were identified. We then identified all cells that used these programs as their top program and visualized program usage within each region of interest via spatial heatmaps.

Data are available at https://osf.io/yndsu.

## Results

### Experiment 1: within-subject yoking

**Figure 1.**
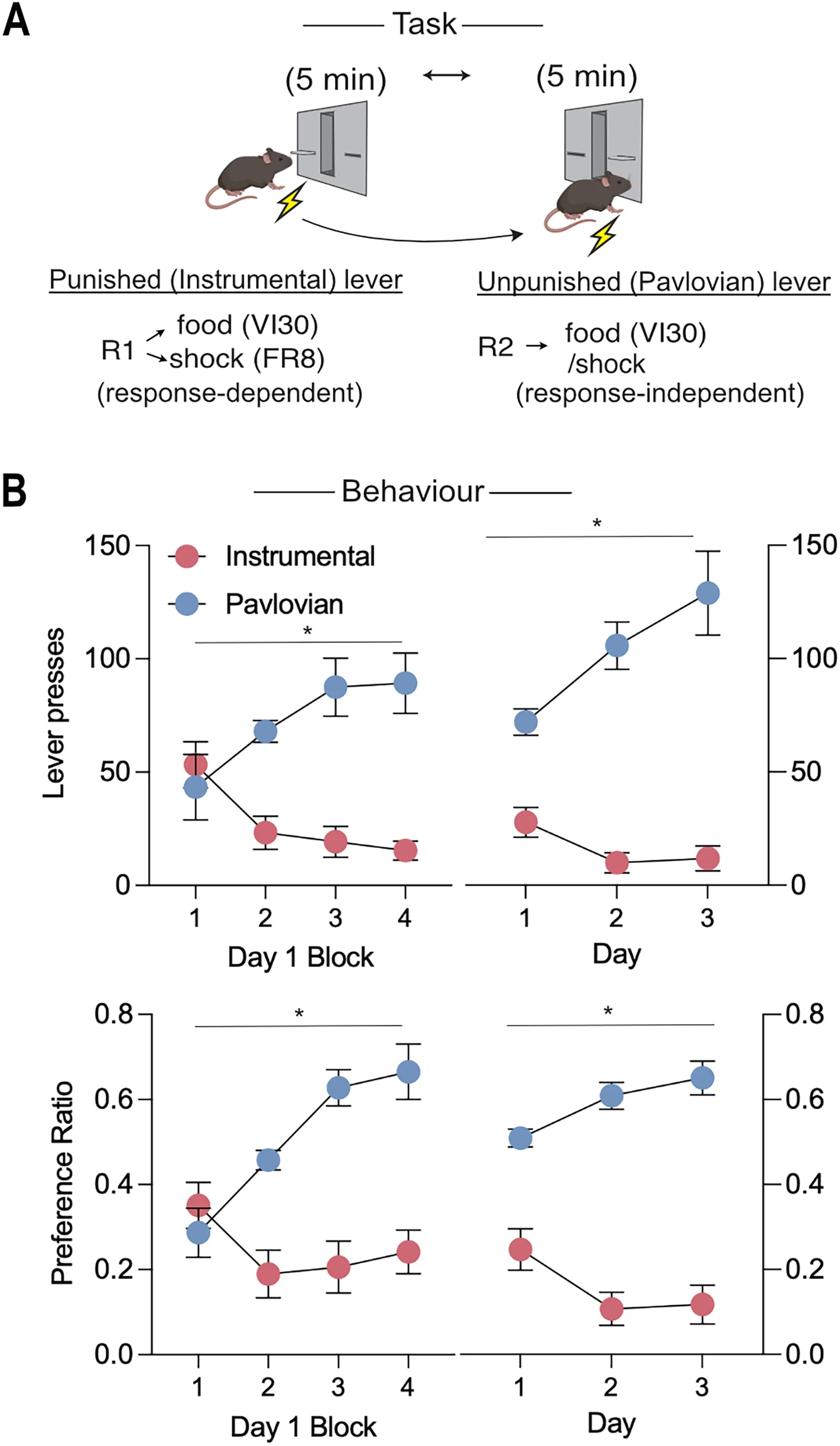
Within-subject yoking: (***A***) Schematic of the within-subject yoking procedure. Mice (*n* = 8) received alternating 5 min access to two levers. On the instrumental lever (*R*1), food was delivered on a VI30 schedule and footshock on an FR8 schedule. For each mouse, shock delivery times from *R*1 were recorded and replayed in a response-independent manner during access to the pavlovian lever (*R*2), which delivered food on a VI30 schedule. This design equates pavlovian contingencies across *R*1 and *R*2 while preserving the instrumental contingency on *R*1. ***B***, Behavioral measures. Mean ± SEM lever-press rates across Day 1 blocks and across days. Pressing was selectively suppressed on the instrumental lever. ***C***, Mean ± SSEM preference ratios computed for each lever separately relative to last day of VI training. *p < 0.05.

We first established the behavioral effects of punishment using a within-subject, two-response task (*n* = 8; Fig. 1*A*). The goal was to assess whether the suppression of punished lever pressing was due to the instrumental response–punisher contingency or to the embedded pavlovian stimulus–outcome contingency. To achieve this, we used a within-subject yoking task (*n* = 8). Here, *R*1 delivered food reward on a VI schedule and a footshock punisher under an FR8 schedule. We recorded timing of punisher delivery and then played these back in a response noncontingent manner to the same animal during access to *R*2. This procedure matches, within the same animal, *R*1 and *R*2 on any pavlovian (i.e., stimulus–outcome) contingencies but separates them on instrumental (i.e., response–outcome) contingency, so that only *R*1 stands in an instrumental relation to shock. If pavlovian stimulus–outcome learning drives suppression, mice should suppress *R*1 and *R*2 equally. In contrast, if instrumental response–outcome learning is driving suppression, mice should suppress *R*1 and not *R*2.

We assessed lever-press rates (Fig. 1*B*). During the first yoked training day, mice suppressed *R*1 relative to *R*2 (*F*_(1,7)_ = 21.993; *p* = 0.002). There was no main effect of block (*F*_(1,7)_ = 0.158; *p* > 0.05), but there was a block-by-response interaction (*F*_(1,7)_ = 18.65; *p* = 0.003) showing that the difference in lever-press rates increased across training. Across the 3 d of yoked training, mice suppressed *R*1 relative to *R*2 (*F*_(1,7) _= 38.632; *p* < 0.001). There was no main effect of day (*F*_(1,7)_ = 5.34; *p* > 0.05), but there was a day-by-response interaction (*F*_(1,7) _= 28.118; *p* = 0.001) showing that the difference in lever-press rates increased across days training.

Next, we assessed lever preference by computing a preference ratio for each lever relative to the last day of VI training (Fig. 1*B*). This preference measure is potentially more sensitive to any stimulus–outcome associations on the pavlovian/unpunished lever. Again, during the first yoked training block, there was a main effect of response (*F*_(1,7)_ = 16.356; *p* = 0.005), no main effect of block (*F*_(1,7)_ = 5.040; *p* > 0.05), and a block-by-response interaction (*F*_(1,7)_ = 17.880; *p* = 0.004). Across the 3 d of yoked training, there was also a main effect of response (*F*_(1,7)_ = 39.458; *p* < 0.001), no main effect of day (*F*_(1,7)_ = 0.061; *p* > 0.05), and a day-by-response interaction (*F*_(1,7)_ = 73.643; *p* = 0.001). Crucially, the daily average preference ratios were ≥0.5, indicating no reduction in preference for the pavlovian/unpunished lever. Taken together, these results demonstrate the causal role of the instrumental response–punisher contingency in controlling behavior.

### Experiment 2: whole-brain Fos

**Figure 2.**
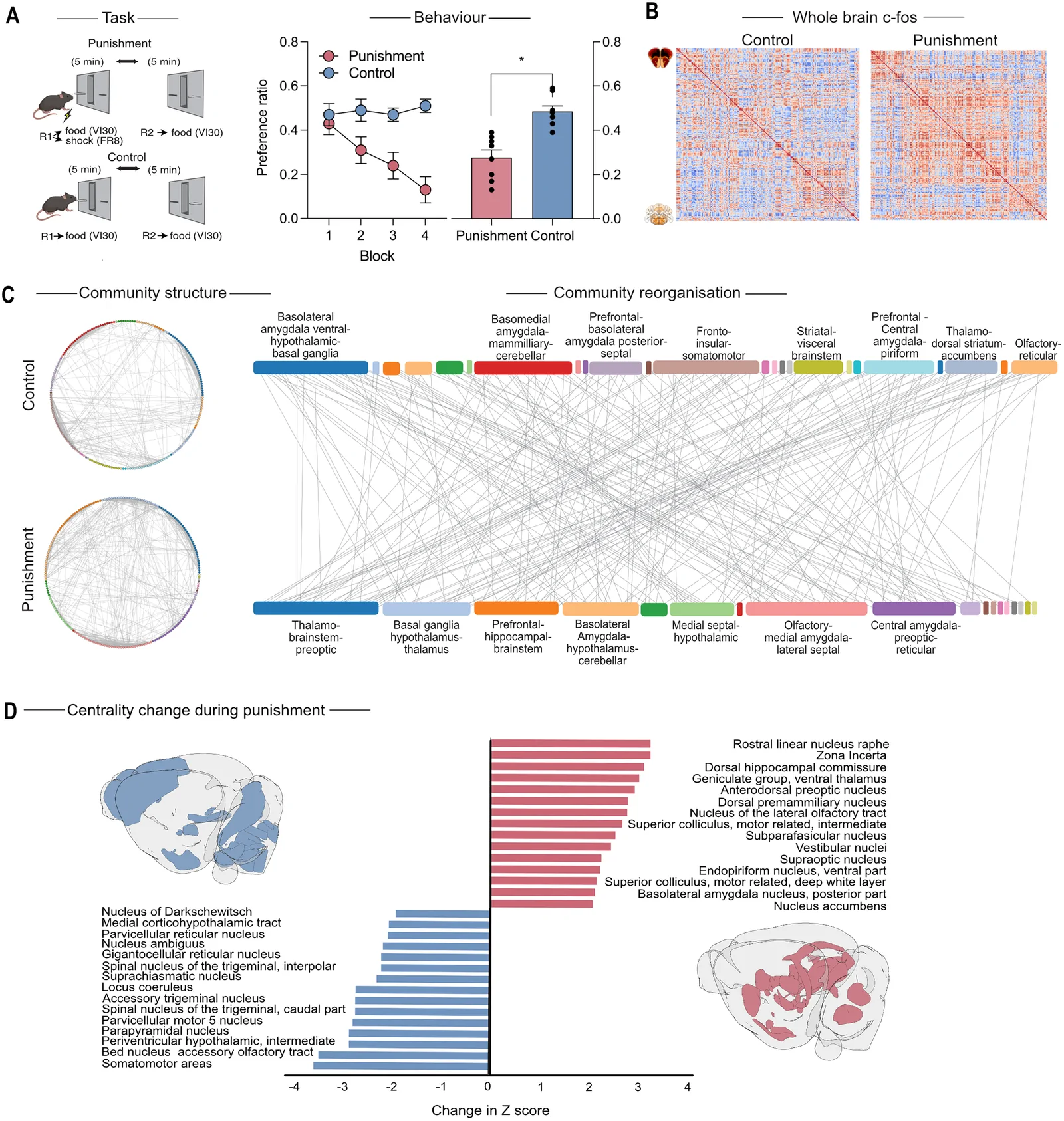
Network analysis of whole-brain c-Fos identifies a distinct Punishment network. ***A***, Experimental design. Punished mice (*n* = 8) were trained with *R*1→punishment (0.3 mA, 0.5 s) + reward and *R*2→reward in alternating 5 min blocks. Control mice (*n* = 8) received *R*1→reward and *R*2→reward. Mean ± SSEM preference ratios are shown across 5 min training blocks and averaged across the session. ***B***, Functional connectivity. Whole-brain c-Fos Pearson’s correlation matrices for Punished and Control groups. ***C***, Community organization. Circle plots illustrating the community structure of brain networks in Punished and Control groups. Each color represents a distinct community of regions that coactivate. The tanglegram links the same brain regions across groups. Each line shows how individual brain regions and their community memberships reorganize following punishment learning. ***D***, Network centrality. Change in normalized degree centrality for the top and bottom 15 brain regions. *p < 0.05.

We next examined the whole-brain correlates of punishment learning. After pretraining where two responses (*R*1, *R*2) were separately reinforced with food on a variable interval (VI) schedule, we implemented punishment (*n* = 8; Fig. 2*A*). Here, one response (*R*1) continued to deliver food reward on VI schedule but also delivered a footshock punisher under a fixed-ratio (FR8) schedule, whereas the alternative response (*R*2) delivered food without shock. Control animals (*n* = 8) experienced the same food contingencies in the absence of punishment. Adaptive avoidance was evident as selective suppression of the punished response (*R*1) relative to the unpunished response (*R*2), which increased steadily over training blocks compared with controls. Analyses of these lever preference ratios showed a main effect of group (*F*_(1,14)_ = 24.16; *p* < 0.001), block (*F*_(1,14)_ = 12.67; *p* = 0.003), and a group × block interaction (*F*_(1,14)_ = 19.5; *p* = 0.001) showing that punishment selectively reduced responding on the punished lever.

To map brain-wide reorganization accompanying punishment learning, we combined tissue clearing, Fos immunolabeling, and light-sheet imaging to quantify activity across 206 brain regions (Fig. 2*B*). Mean and SEM c-Fos densities (cells/mm^3^) are shown in Figure S2. We tested group differences across the 206 brain regions and controlled for multiple comparisons using the Benjamini–Hochberg false discovery rate procedure. Thirteen regions showed evidence of group differences at uncorrected *p* < 0.05, including the ventromedial hypothalamic nucleus, epithalamus, perifornical nucleus, paratrigeminal nucleus, suprachiasmatic nucleus, caudal spinal nucleus of the trigeminal, septohippocampal nucleus, caudoputamen, olivary pretectal nucleus, septofimbrial nucleus, dorsal nucleus of the inferior colliculus, dorsal column nuclei, and sublaterodorsal nucleus. However, none of these effects survived FDR correction across the full set of regional comparisons; the smallest FDR-adjusted *p* value was observed for the ventromedial hypothalamic nucleus (*q* = 0.225).

We next tested whether punishment learning was reflected in the organization of coordinated activity across regions (Pessoa, 2022). To do this, we treated each brain region as a node and the correlation in Fos expression between regions as an edge, allowing us to construct brain-wide Fos correlation networks for punished and control mice. Correlation matrices (Fig. 2*B*) were thresholded at |*r*| ≥ 0.8, binarized, and used to generate coactivation networks (Fig. S3).

Both Control and Punishment networks exhibited modular, small-world topology (*σ*_Control_ = 5.55; *σ*_Punishment_ = 4.21; Fig. S3), indicating that the broad organizing principles of brain networks were preserved after punishment learning (Bullmore and Sporns, 2009; 2012; Basset and Sporns, 2017; Faskowitz et al., 2022). However, the composition of these modules changed substantially. Community detection identified 20 communities in the Control network and 17 communities in the Punishment network. Here, communities refer to groups of brain regions whose activity covaried more strongly with each other than with regions outside the group (Fig. 2*C*). Control and Punishment community structure differed markedly (Fig. 2*C*; Dataset S1). Quantitative comparison of the two partitions showed very low similarity, with an adjusted Rand index of 0.025 and normalized mutual information of 0.254. These low values indicate that punishment learning did not simply strengthen or weaken connections within a fixed network architecture. Instead, many regions were reassigned into different communities (Fig. 2*C*). Notably, under punishment, a community (“prefrontal–hippocampal–brainstem”) emerged linking prefrontal regions, including anterior cingulate, prelimbic, infralimbic, and orbital cortices, with limbic regions including the hippocampus, and brainstem neuromodulatory structures including the superior colliculus, locus ceruleus, and laterodorsal tegmental nucleus.

To identify which brain regions were most influential within the coactivation network (Fig. 2*D*), we compared node centrality between Punishment and Control networks. Centrality provides an index of hub-like influence: regions with higher centrality are more strongly embedded in network communication, whereas regions with lower centrality contribute less to the overall functional architecture. Punishment learning produced a marked redistribution of regional influence. Regions associated with behavioral automaticity, including the somatomotor cortex, decreased in centrality during punishment, suggesting reduced dominance of motor-related architecture. In contrast, regions across the amygdala, subthalamic–hypothalamic zone, and midbrain increased in centrality during punishment. These included the BLA, ZI, and RLi, which emerged as hub nodes in the Punishment network.

### Experiment 3: spatial transcriptomics

**Figure 3.**
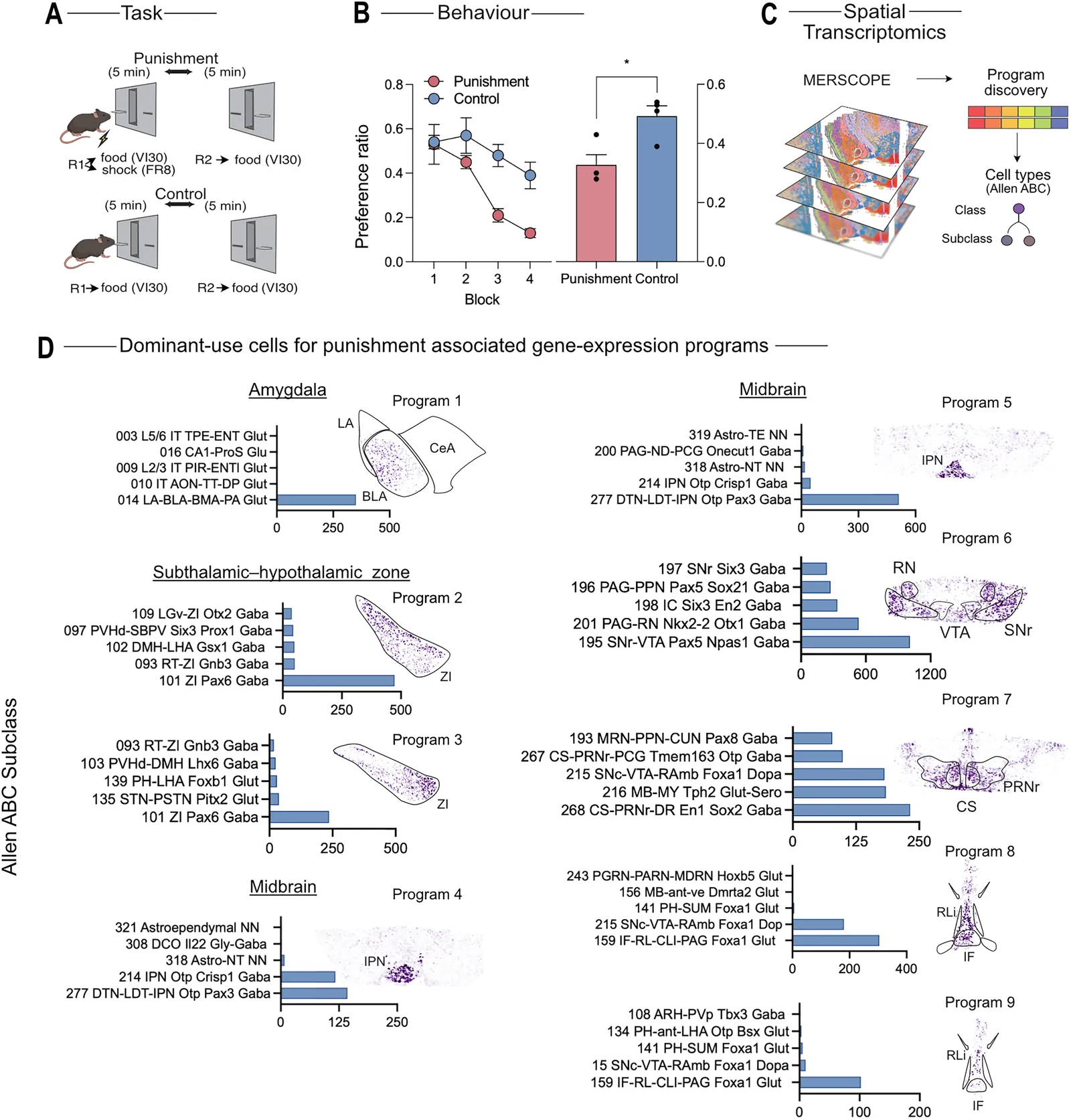
Spatial transcriptomics identifies region- and cell-type-specific gene-expression programs engaged by punishment learning. ***A***, Task. Mice (*n* = 4) were trained with *R*1→punishment (0.3 mA, 0.5 s) + reward and *R*2→reward in alternating 5 min blocks. Control mice (*n* = 4) received *R*1→reward and *R*2→reward. ***B***, Behavior. Mean ± SSEM preference ratios across 5 min training blocks and averaged across the session (note: 2 animals in the Punished group had preference ratios, 0.3). ***C***, Spatial transcriptomics workflow. Tissue sections from the amygdala, subthalamic–hypothalamic zone, and midbrain were imaged using the MERSCOPE platform. Gene-expression programs were derived by cNMF and mapped to cell types defined by the Allen Brain Cell (ABC) Atlas. ***D***, Punishment-specific gene-expression programs. Top program usage by brain region and Allen ABC subclass and coronal sections show spatial heatmaps of cells with top program usage. *p < 0.05 (***B***). Data are mean ± SSEM (***B***) and total cell counts (***D***).

Having identified the amygdala, subthalamic–hypothalamic zone, and ventral midbrain tegmentum as key hubs in the Punishment network, we next asked which molecularly defined cell populations within these regions were associated with punishment learning. Mice were trained in the within-subject punishment task, with separate Punished and Control groups (Fig. 3*A*). As expected, Punished mice selectively suppressed the punished action across training, whereas Control mice maintained responding (group, *F*_(1,6)_ = 11.52; *p* = 0.015; block, *F*_(1,6)_ = 46.37; *p* < 0.001; group × block, *F*_(1,6)_ = 9.76; *p* = 0.020; Fig. 3*B*). After training, we used the Vizgen MERSCOPE platform to profile spatial gene expression across coronal sections containing the amygdala, subthalamic–hypothalamic zone, and ventral midbrain tegmentum (Fig. 3*C*).

We used cNMF to identify transcriptional programs recurring across cells within each region (Kotliar et al., 2019). A gene-expression program refers to a coordinated pattern of coexpressed genes shared by a subset of cells. This approach allowed us to move beyond single marker genes and identify broader molecular states associated with punishment learning. For each cell, cNMF estimated the degree to which that cell expressed each program, yielding a cell-level program usage score. Thus, our analysis focused not on individual differentially expressed genes but on reproducible combinations of genes that defined punishment-associated transcriptional states within spatially defined neuronal populations. We defined punishment-associated programs as programs that were reproducibly detected in every Punished animal but were not recapitulated in Control animals, as determined by similarity analyses and leave-one-out resampling. This identified nine punishment-associated programs: one in the amygdala, two in the subthalamic–hypothalamic zone, and six in the ventral midbrain tegmentum. We then used MapMyCells to assign cells to Allen Brain Cell Atlas reference subclasses, allowing each punishment-associated program to be linked to molecularly defined neuronal populations.

We analyzed the relationship between punishment-associated programs and cell subclasses in two complementary ways. First, for the spatial analysis, we identified cells in which each punishment-associated program was the dominant program and mapped their anatomical distribution across each region (Fig. 3*D*). This analysis identifies the cells most strongly characterized by each punishment-associated transcriptional state. Second, we quantified mean usage of each punishment-associated program across Allen ABC subclasses and reported the top-ranked subclasses, together with the number of detected cells in each subclass (Dataset S2). This analysis captures broader cell-class-level recruitment of each program, including cases in which a program was expressed across a population but was not the dominant program in every individual cell. So, the dominant-program analysis identifies the spatial distribution of cells most strongly characterized by a punishment-associated transcriptional state, whereas the mean-usage analysis identifies the broader Allen ABC subclasses that carry that transcriptional signal across the population. Together, these analyses distinguish the spatial distribution of strongly program-positive cells from the broader neuronal subclasses that carry each punishment-associated transcriptional signal.

The spatial analysis (Fig. 3*D*; Dataset S3) revealed a heterogeneous molecular architecture across the Punishment network. In the amygdala, a single punishment-associated program was detected in BLA glutamatergic neurons distributed across the BLA. This program was characterized by top-weighted genes associated with synaptic plasticity and neuromodulatory signaling, including *Bdnf*, *Cckbr*, *Sstr2*, *Oprd1*, and *Ntng2*. In the subthalamic–hypothalamic zone, two punishment-associated programs were detected in the ZI. These programs occupied partially distinct spatial territories, with Program 2 enriched in anterior ZI and Program 3 enriched more posteriorly. Both programs were most strongly associated with Pax6-expressing GABAergic ZI neurons, a population implicated in multisensory integration, anxiety, and motivational control.

The ventral midbrain tegmentum showed the greatest molecular diversity. Programs 4 and 5 were centered in the interpeduncular nucleus and were associated with Otp-Pax3 and Otp-Crisp1 GABAergic populations. Programs 6 and 7 involved GABAergic populations marked by *Pax5*, *Otx1*, and *En1*, as well as *Foxa1* dopaminergic neurons, spanning the ventral tegmental area, substantia nigra reticulata, red nucleus, and pontine reticular nucleus. Programs 8 and 9 were centered near the RLi and interfascicular region and were associated with *Foxa1* glutamatergic and dopaminergic populations linked to mesocorticolimbic and mesothalamic pathways.

Together, these findings show that punishment learning is associated with a distributed but spatially structured set of transcriptional programs across the Punishment network, with region-specific molecular states in BLA glutamatergic neurons, ZI GABAergic neurons, and multiple ventral midbrain populations, especially GABAergic populations.

### Experiment 4: chemogenetic inhibition

Our whole-brain Fos and spatial transcriptomic data identify the BLA, ZI, and RLi as key nodes in a Punishment network. However, these are descriptive and correlational. To assess the causal roles of hub brain regions identified in the Punishment network, we first employed computational lesions to quantify their contribution to global network structure and integration. We performed in silico deletions of the three hub regions, BLA, ZI, and RLi, either individually or in combination to examine what effect, if any, lesions of these hub regions would have on network function and properties. In this graph-theoretic method, individual nodes are computationally removed and the effects on global network properties (connectivity, integration, and efficiency) are measured, isolating each region's structural contribution. Deletion of any single hub or triple deletion of the three hubs had minimal effects on basic network properties, including the size of the LCC, total number of edges, average node degree, and shortest path length, measures capturing direct connectivity and optimal routing (Fig. S3). This relative insensitivity is expected for small-world, modular networks, in which local redundancy and alternative pathways preserve basic topological features despite focal perturbations (Bullmore and Sporns, 2009).

Rather, focal deletion selectively reduced global communicability, a metric that captures the efficiency with which signals propagate through all possible network walks, including indirect and longer-range routes (Bullmore and Sporns, 2009). Deletion of each hub individually produced a reduction in communicability, identifying BLA, ZI, and RLI as three contributors to network-wide information flow rather than to local connectivity alone (Fig. S3). That each hub degraded communicability when removed in isolation provided the direct rationale for testing every region's causal contribution to punishment learning with single-region chemogenetic inhibition.

Simultaneous deletion of all three regions reduced communicability further, to a degree exceeding any single deletion (Fig. S3). To test the specificity of this observation, we generated a null distribution by deleting 5,000 randomly selected triples of nodes from the Punishment network. The vast majority of random triplet deletions produced only modest reductions in communicability. In contrast, simultaneous removal of the BLA, ZI, and RLi caused a 79.97% reduction in communicability that lay significantly outside the null distribution (*p* = 0.0001; *N* = 5,000), confirming that the observed disruption cannot be explained by removal of three nodes per se. Together, these analyses identify BLA, ZI, and RLi as hubs whose individual and coordinated integrity is critical for maintaining network-wide information flow during punishment learning, providing the rationale for concurrent in vivo silencing of these three regions.

**Figure 4.**
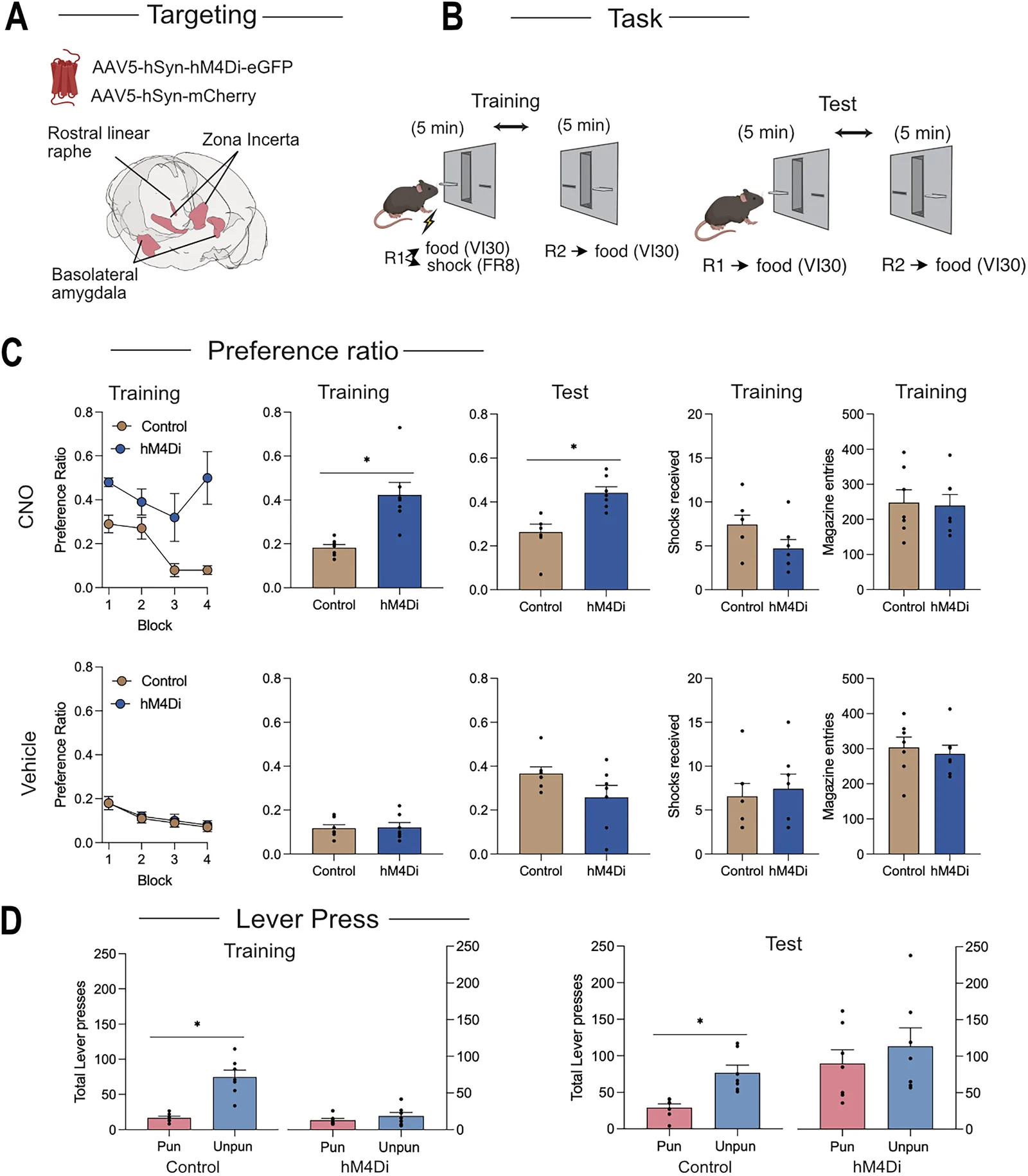
Triple chemogenetic silencing identifies hub-node roles in punishment. ***A***, Viral targeting. Mice received AAVs encoding the inhibitory DREADD hM4Di or control mCherry in the BLA, ZI, and RLi. ***B***, Experimental design. Mice were trained with *R*1→punishment (0.3 mA, 0.5 s) + reward and *R*2→reward in alternating 5 min blocks. Intraperitoneal injections of CNO (3 mg/kg) or vehicle were administered before training, and testing occurred 48 h later. ***C***, Mean ± SSEM preference ratios across training blocks, overall training, and test sessions as well as shocks received and magazine entries (hM4Di *n* = 7; Control *n* = 7). ***D***, Mean ± SSEM lever-press rates during CNO training and test. *p < 0.05.

To test these network-level predictions in vivo, we used multisite chemogenetic inhibition during punishment learning (Fig. 4*A*). Mice (*n* = 7) received infusions of the inhibitory DREADD AAV5-hSyn-hM4Di into the amygdala (targeting BLA), subthalamic–hypothalamic zone (targeting ZI), and ventral midbrain tegmentum (targeting RLi) concurrently, with controls (*n* = 7) receiving AAV5-hSyn-mCherry. Figure 6 shows placement maps for this AAV expression. Mice were trained in the within-subject punishment task as before. Prior to punishment training, they received injections of the DREADD ligand CNO (3 mg/kg) and vehicle. Mice were tested 48 h later after training in the absence of punishment.

Mean and SEM lever preference ratios are shown in Figure 4*C*. Triple silencing impaired punishment learning as shown by impaired preference in the hM4Di group during both punishment training and test that was specific to silencing during punishment training via CNO. During punishment training, there was a main effect of Group (Control vs hM4Di; *F*_(1,12)_ = 13.24; *p* = 0.003), a main effect of injection (Vehicle vs CNO; *F*_(1,12)_ = 32.88; *p* < 0.001), and a group × injection interaction (*F*_(1,12)_ = 14.77; *p* = 0.002). Follow-up comparison showed a significant difference between hM4Di and Controls during CNO (*t*_(12)_ = 4.06; *p* < 0.011) but not vehicle session (*t*_(12)_ = −0.21; *p* > 0.05). During the test, there was no main effect of Group (*F*_(1,12)_ < 1; *p* > 0.05) or Injection (*F*_(1,12)_ = 1.49; *p* > 0.05), but there was a Group × Injection interaction (*F*_(1,12)_ = 19.89; *p* = 0.001). Follow-up comparison again showed a significant difference between hM4Di and Controls after injection of CNO (*t*_(12)_ = 4.06; *p* < 0.011) but not vehicle (*t*_(12)_ = 1.76; *p* > 0.05).

During punishment training under CNO, there were no differences between Control and hM4Di groups in the number of punishers received (*t*_(12)_ = 1.843; *p* = 0.09) or in magazine entries (*t*_(12)_ = 0.179; *p* = 0.861; Fig. 4*C*). There were also no differences between groups in these measures during punishment training under vehicle (punishers received, *t*_(12)_ = 0.38; *p* = 0.708; magazine entries, *t*_(12)_ = 0.87; *p* = 0.401). This suggests that the effects of chemogenetic silencing were selective to learning rather than causing effects on general performance such as sensory or motivational loss. Inspection of mean and SEM lever-pressing rates further confirmed that these brain regions are necessary for adaptive punishment avoidance rather than nonspecific behavioral inhibition (Fig. 4*D*). Whereas Control animals suppressed responding on the punished lever relative to the unpunished lever during training (*t*_(6)_ = 6.6; *p* < 0.001) and test (*t*_(6)_ = 5.9; *p* < 0.001), hM4Di animals did not (training, *t*_(6)_ = 1.59; *p* = 0.162; test, *t*_(6)_ = 1.61; *p* = 0.159). So, chemogenetic inhibition impaired the response-selective learning that characterizes punishment.

**Figure 5.**
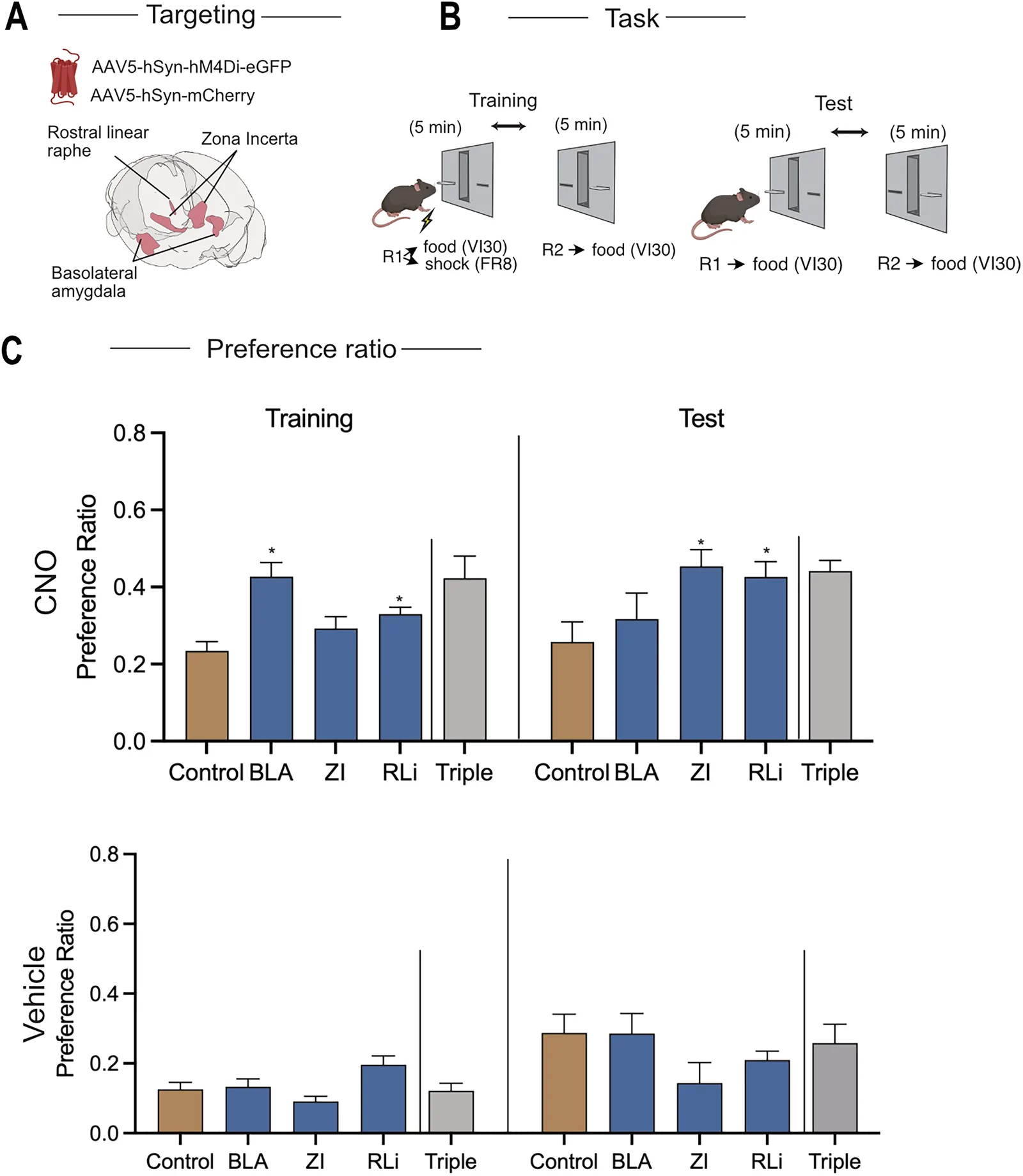
Single chemogenetic silencing identifies hub-node roles in punishment. ***A***, Viral targeting. Mice received AAVs encoding the inhibitory DREADD hM4Di in the BLA (*n* = 8) or ZI (*n* = 7) or RLi (*n* = 8) or control mCherry (*n* = 12). ***B***, Experimental design. Mice were trained with *R*1→punishment (0.3 mA, 0.5 s) + reward and *R*2→reward in alternating 5 min blocks. Intraperitoneal injections of CNO (3 mg/kg) or vehicle were administered before training, and testing occurred 48 h later. ***C***, Mean ± SSEM preference ratios across training and test. *p < 0.05.

**Figure 6.**
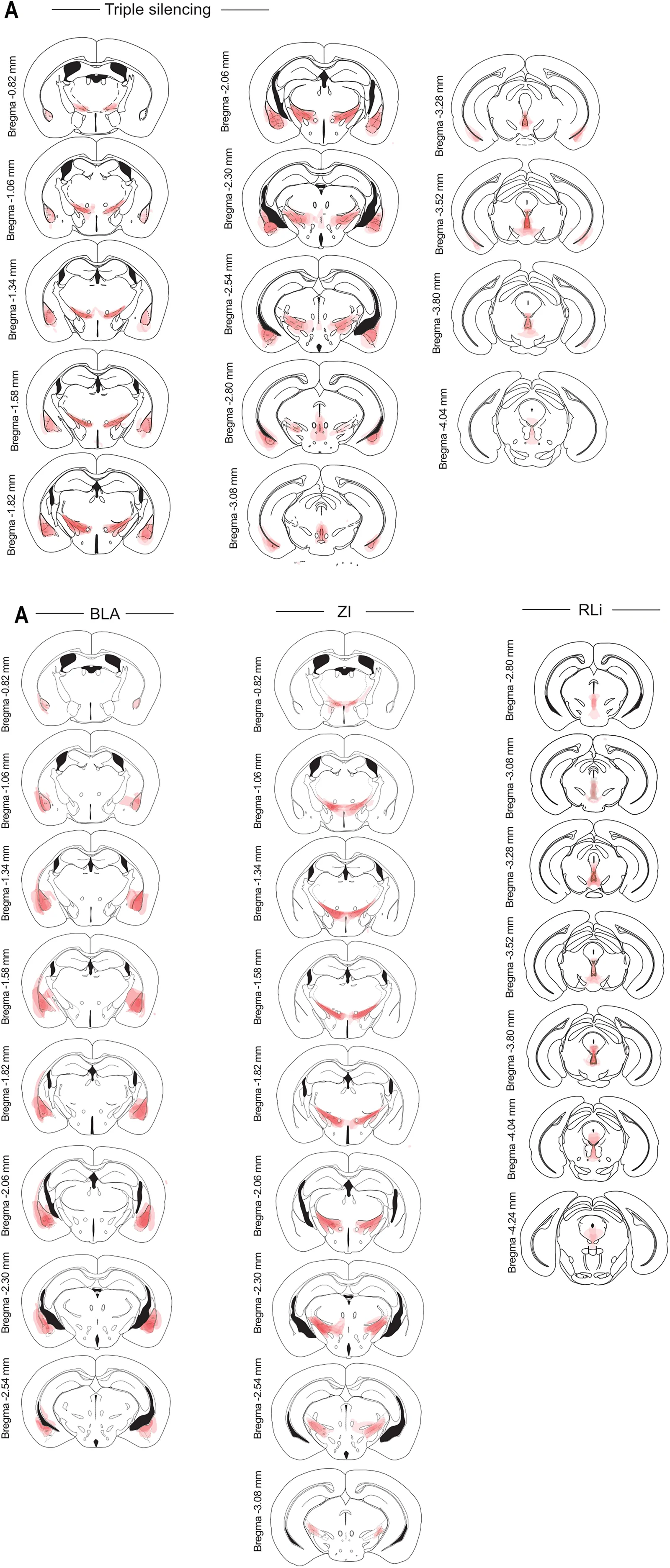
Histological verification of hM4Di expression. Location of hM4Di expression in the amygdala, subthalamic–hypothalamic zone, and ventral midbrain tegmentum on Paxinos and Franklin atlas. Triple expression is shown in ***A***; single-region expression is shown in ***B***.

We next asked whether chemogenetic silencing of any single brain region could account for this effect. Using the same hM4Di inhibition and behavioral training approach, we targeted individually the BLA (*n* = 7), subthalamic–hypothalamic zone centered on the ZI (*n* = 8), or ventral midbrain tegmentum centered on RLi (*n* = 8; Fig. 5*A*). A control group (*n* = 12) was comprised of mice that received AAV5-hSyn-eGFP injections into one of these regions alone (*n* = 4 per region). Figure 6 shows placement maps for this AAV expression. Mice were trained in the within-subject punishment task (Fig. 5*B*). Prior to punishment training, they received injections of the DREADD ligand CNO (3 mg/kg) and vehicle. Mice were tested 48 h later after training in the absence of punishment.

Mean and SEM lever preference ratios are shown in Figure 5*C*. Data from triple silencing data are reproduced from Figure 4 for comparison. During punishment training, there was a main effect of Group (*F*_(3,31)_ = 5.101; *p* = 0.006), a main effect of injection (Vehicle vs CNO; *F*_(1,31)_ = 280.225; *p* < 0.0001), and a Group × Injection interaction (*F*_(3,31)_ = 14.572; *p* = <0.0001). During the test, there was no main effect of Group (*F*_(3,31)_ = 0.248; *p* = 0.862), but there was a main effect of training injection (Vehicle vs CNO; *F*_(1,31)_ = 18.220; *p* < 0.0002) and a Group × Injection interaction (*F*_(3,31)_ = 6.069; *p* = <0.001). Follow-up pairwise comparisons versus controls showed an effect of BLA inhibition during training (*F*_(1,31)_ = 25.149; *p* < 0.0001) that did not carry-over to the test (*F*_(1,31)_ = 0.523; *p* = 0.547), indicating that chemogenetic silencing centered on BLA impaired within-session but not between-session punishment. In contrast, silencing centered on ZI had no effect during training (*F*_(1,31)_ 2.445; *p* = 0.128) but did lead to later impairment on the test (*F*_(1,31)_ = 7.44; *p* = 0.010). Silencing centered on RLi had a modest effect during training (*F*_(1,31)_ = 6.675; *p* = 0.015) and test (*F*_(1,31)_ = 5.429; *p* = 0.026). Together, these findings show that individual region silencing did not recapitulate the impairment caused by triple silencing. Instead, these findings show a functional dissociation within the Punishment network: BLA is required for the short-term, within-session suppression of punished actions, ZI activity is required for the retention of punishment learning across sessions, and RLi contributes to both phases more modestly.

## Discussion

Here we show that suppression of a punished action depends on the response–punisher contingency, is accompanied by reorganization of coordinated activity across the brain, recruits spatially structured transcriptional programs in key network hubs, and requires the function of the amygdala, subthalamic–hypothalamic zone, and ventral midbrain tegmentum. Together, these findings suggest that punishment learning depends on a distributed network in which different brain regions make complementary contributions to adaptive suppression of punished actions (Pessoa, 2022).

Our within-subject yoking procedure separated instrumental response–punisher learning from pavlovian stimulus–shock learning (Boe and Church, 1968; Church, 1988). Because the same animals received the same pattern of shock exposure during access to both levers, suppression driven by chamber cues, lever- stimulus cues, or general fear should have reduced responding on both levers. Instead, mice selectively suppressed the response that caused shock while maintaining responding on the yoked response that was accompanied by the same shocks but did not cause them. This demonstrates that response suppression was controlled by the instrumental action–punisher contingency. Many behavioral effects of aversive outcomes can be explained by pavlovian conditioned suppression, general motivational disruption, or nonspecific fear (Bolles et al., 1975, 1980). Here, suppression was response selective, contingency dependent, and therefore instrumental (Mackintosh, 1983).

Region-wise Fos analyses did not reveal any individual brain region that survived correction for multiple comparisons. The punisher was mild and, as mice learned punishment and selectively suppressed punished lever pressing, differences in experience relative to controls decreased. Instead, network analyses showed reconfiguration of brain-wide Fos correlation structure. Both control and Punishment networks retained modular, small-world organization. However, community composition changed markedly. There was a reallocation of regional participation mirroring principles described for task switching, cognitive control, and adaptive reconfiguration under changing demands (Cocchi et al., 2013; Braun et al., 2015; Shine et al., 2016). Punishment learning reduced the influence of regions involved in behavioral automaticity (e.g., somatomotor cortex; Killcross and Coutureau, 2003; Balleine and O’Doherty, 2010) while increasing centrality in regions spanning the amygdala, subthalamic–hypothalamic zone, and ventral midbrain tegmentum. The emergence of the BLA, ZI, and RLi as hub nodes suggests that punishment learning shifts the brain from a motor-dominant architecture toward a network more strongly organized around aversive valuation (Namburi et al., 2015; Kim et al., 2016; Tye, 2018) and sensory motor integration (Wang et al., 2020), consistent with the behavioral requirement of the task.

Experiment 3 asked which molecularly defined cell populations within Punishment network hubs were associated with learning. Spatial transcriptomics revealed punishment-associated gene-expression programs in BLA glutamatergic neurons, ZI GABAergic neurons, and multiple ventral midbrain populations. In the BLA, a single punishment-associated program was detected in glutamatergic neurons. This is consistent with the established role of BLA excitatory populations in valence coding (Namburi et al., 2015; Kim et al., 2016; Tye, 2018) and in using outcome value to guide action selection (Balleine and Killcross, 2006; Balleine, 2019; Piantadosi et al., 2026). In the ZI, two punishment-associated programs were associated with Pax6-expressing GABAergic neurons and occupied distinct anterior–posterior niches. This suggests that punishment learning may recruit more than one ZI microcircuit. ZI GABAergic populations have been implicated in aversion, sensory integration, and behavioral flexibility (Zhou et al., 2018; Zhao et al., 2019; Wang et al., 2020; Li et al., 2021), all of which are relevant to the selective suppression required in this task. The ventral midbrain tegmentum showed the greatest molecular diversity. IPN-centered programs involved Otp-Pax3 and Otp-Crisp1 GABAergic populations, consistent with the role of IPN-related circuitry in regulating responses to aversive events (Liang et al., 2024). Other programs involved Pax5, Otx1, and En1 GABAergic populations, together with Foxa1 dopaminergic neurons, spanning regions linked to action selection and motor control, including the VTA, SNr, red nucleus, and pontine reticular nucleus (Costa and Schoenbaum, 2022). Programs centered near the RLi and interfascicular region encompassed Foxa1 glutamatergic and dopaminergic neurons linked to aversion signaling via mesocorticolimbic and mesothalamic pathways (Yamaguchi et al., 2011; Root et al., 2018, 2020; Barbano et al., 2020). These findings are especially notable given recent evidence that precisely timed GABAergic input to dopamine neurons is critical for punishment learning (Lefner and Moghaddam, 2025; Tan et al., 2026).

The final question was whether regions identified by the network analysis were necessary substrates for learning and avoidance. Experiment 4 provided causal evidence that the BLA, ZI, and RLi are required for adaptive punishment avoidance. In silico deletion analyses predicted that these regions support network-wide communicability. In vivo chemogenetic silencing supported this prediction. Triple silencing of BLA, ZI, and RLi impaired punishment learning specifically by preventing selective suppression of the punished response during training and impaired later test performance, without reducing magazine entries or altering the number of punishers received. These argue against a nonspecific explanation. Instead, the deficit was specific to the response-selective learning required for punishment. The single-region silencing experiments revealed a further dissociation at different timescales. BLA inhibition impaired within-session suppression during punishment training but did not impair later test performance. This suggests that BLA activity is especially important for immediate or working memory use of punishment information. In contrast, ZI inhibition spared within-session suppression but impaired later test performance, suggesting a role in the consolidation of punishment learning. RLi inhibition produced more modest impairments across both training and test, consistent with a contribution to both acquisition and expression. Importantly, no single-region manipulation reproduced the magnitude of impairment caused by triple silencing. This supports the conclusion that punishment learning depends on distributed network function rather than the isolated operation of any single region (Pessoa, 2022).

### Methodological considerations

The within-subject yoking procedure tested whether embedded stimulus–shock relations or shock exposure were sufficient to suppress responding. They were not. The subsequent experiments were therefore designed to identify the mechanisms of this response-selective punishment learning rather than to repeat the pavlovian–control comparison in each experiment. This is an important consideration for interpretation. It is possible that pavlovian stimulus–shock associations contributed to some elements of the observed Fos or transcriptional signatures, especially in circuits sensitive to shock or arousal. However, such associations cannot explain the response-selective behavioral suppression observed here. In addition, chemogenetic inhibition of the identified hubs impaired selective suppression of the punished action. It will be important for future work to continue to address and control for stimulus–shock relations, and the findings reported here are best interpreted as identifying mechanisms recruited during instrumental punishment learning while recognizing that this learning always occurs within a broader aversive context.

Whole-brain Fos analysis provides a snapshot of activity associated with the behavioral state at the time of tissue collection. Fos is a powerful marker for identifying candidate brain-wide activity patterns (Kimbrough et al., 2020; Hansen et al., 2021; Roland et al., 2023; Stefaniuk et al., 2023), but it is an indirect and temporally integrated measure. Regional Fos analyses were limited by the number of animals relative to the number of regions tested. Although 13 regions showed nominal group differences, none survived FDR correction. Network analyses depend on common choices to enable interpretable graph construction (Terstege and Epp, 2022), but these may influence estimates of community structure and centrality (Bullmore and Sporns, 2009; Basset and Sporns, 2017). Moreover, these are functional not anatomically defined networks. Nonetheless, the demonstrated convergence between network analyses and effects of chemogenetic inhibition strengthens confidence in the relevance of this analysis.

Spatial transcriptomic analyses identified candidate cell types within Punishment network hubs. By combining cNMF with Allen Brain Cell Atlas mapping, we linked punishment-associated transcriptional programs, estimated from a targeted MERSCOPE gene panel, to defined neuronal populations (Kotliar et al., 2019). These findings should be interpreted as identifying candidate cell populations carrying coordinated transcriptional states within the limits of the assayed gene panel rather than as defining genome-wide programs, individual causal genes, or the necessity of those populations. Future experiments will be needed to test whether manipulating these specific cell populations or the transcriptional programs they express alters punishment learning.

For chemogenetic inhibition, we did not counterbalance the order of CNO and vehicle injection. The impact on interpretation is unclear. The effects of CNO depended on which and how many regions were silenced, arguing against nonspecific effects. Regardless, future work should address this. Also, such inhibition provides causal evidence but lacks the temporal precision needed to isolate specific learning processes. CNO-induced hM4Di inhibition spans the training session and post-training interval, so the observed deficits could reflect disruption of several processes. The single-region dissociations provide clues, but temporally precise approaches using calcium imaging or optogenetic manipulation will be needed in future work.

### Conclusions

Punishment learning is essential for adaptive behavior because it allows animals to stop actions that produce harm while maintaining other rewarded actions. We show that this form of learning is not explained by shock exposure, pavlovian fear, or activation of a single brain region. Instead, punishment learning depends on the response–punisher contingency, reorganizes brain-wide activity networks, recruits spatially structured transcriptional programs in specific neuronal populations, and requires the function of the amygdala, subthalamic–hypothalamic zone, and ventral midbrain tegmentum.
